# Supplementary material for: Diet-microbiome interactions promote enteric nervous system resilience following spinal cord injury
Source: NPJ Biofilms Microbiomes. 2024 Aug 29;10:75. doi: 10.1038/s41522-024-00556-y (PMC11362535; doi:10.1038/s41522-024-00556-y)
Supplement: Supplementary file 1 — Supplementary Figures 1–3 [file 41522_2024_556_MOESM1_ESM.pdf]

Supplementary Figure Legends

Supplementary Figure 1, Related to Figure 2. Cohort-dependent impacts on SCI-triggered dysbiosis.
16S sequencing results from an independent cohort of male mice. **A, B,** Pre-surgery to end point (14-dpi) changes in fecal microbiome 16S rRNA alpha diversity represented by Chao1(**A**) and Shannon (**B**) diversity measures. **C**, Principal component analysis (PCA) plot representing changes in overall community structure of fecal microbiomes. **D**, Microbial composition barplot, order level, of fecal microbiomes at 14-dpi. Asterisks in (**A)** and (**B)** represent a significant decrease in alpha diversity from 0-dpi to 14-dpi in the sham group. Each circle represents the average of all mice in a group for all but **C**, where each circle represents an individual mouse. N=3-8. * $P < 0.05$. Data are shown as mean ± SEM. Statistically significant differences were determined by repeated measures 2-way ANOVA with post-hoc Sidak's multiple comparison test to compare temporal changes within groups.

Supplementary Figure 2, Related to Figure 3. Injury and diet-associated microbes are not sufficient to trigger NBD.
A-C, Heatmap of cytokines present in proximal colon tissue lysate from male and female uninjured germ-free mice that were colonized with either sham-derived or SCI-veh-derived fecal microbiomes (**A**), as assessed by multiplexed ELISA, with significant increases in CXCL1 (**B**) and IL-12p70 (**C**). **D-G**, Heatmap of serum metabolic markers, as assessed by multiplexed ELISA (**D**), with significant decreases in C-peptide (**E**), TNF (**F**), and ghrelin (**G**). **H-M**, Heatmap of serum metabolic markers in male mice with laminectomy (Sham) or injury (SCI-veh), as assessed by multiplexed ELISA (**H**), with significant decreases in C-peptide (**I**), TNF (**J**), glucagon (**K**), GLP-1 (**L**), and leptin (**M**). Each data point represents one mouse. Squares represent ex-GF males, hexagons represent ex-GF females, and circles represent SPF males. N=19-21 (**A-C**), N=14-15 (**D-G),** N=11-14 (**H-M**). * $P < 0.05$, ** $P < 0.01$, *** $P < 0.001$, **** $P < 0.0001$. Data are shown as mean ± SEM. Statistically significant differences were determined by two-tailed unpaired t-test.

Supplementary Figure 3, Related to Figure 4. SCFA-induced IL-10 signaling is necessary for SCI recovery.
A, Heatmap of cytokines, assessed by multiplexed ELISA, in proximal colon tissue lysate from male wild-type mice with laminectomy (Sham), SCI with standard diet (SCI-vehicle), and SCI with inulin-supplemented diet (SCI-inulin). **B**, Concentration of colonic IL-1β by ELISA. **C-E,** Endpoint quantification of indicated short-chain fatty-acids from fecal pellets of male IL10rb KO mice with SCI (SCI-veh) or inulin-supplemented diet (SCI-inulin). **F**, Quantification of colonic free fatty acid receptor 2 (FFAR2) levels, by western blot, in male IL10rb KO mice with SCI. **G**, Heatmap of cytokines, assessed by multiplexed ELISA, in serum from male wild type mice with SCI treated with either vehicle (SCI-veh), tripropionin (SCI-TRP), or tributyrin (SCI-TRB). **H**, Concentration of serum IL-6 by ELISA. **I-K,** Endpoint quantification of indicated short-chain fatty-acids from serum of male mice with SCI, treated with vehicle (Veh), tripropionin (TRP), or tributyrin (TRB). Each data point represents one mouse. N=11-17 (**A, B**), N=7-10 (**C-F**), N=11-13 (**G, H**), N=5-6 (**I-K**). * $P < 0.05$, ** $P < 0.01$. Data are shown as mean ± SEM. Statistically significant differences were determined by ordinary one-way ANOVA with post-hoc Tukey's (**A, B**) or Dunnett's (**G, K**) multiple comparison test. Or by two-tailed unpaired t-test (**C-F**).

Supplementary Figure 1. Cohort-dependent impacts on SCI-triggered dysbiosis

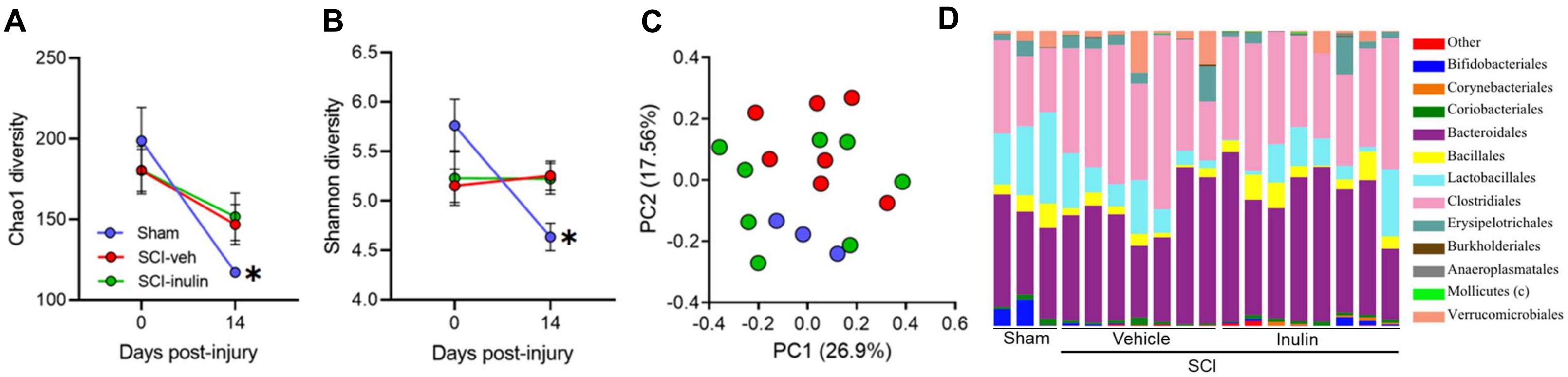

Supplementary Figure 2. Injury and diet-associated microbes are not sufficient to trigger NBD

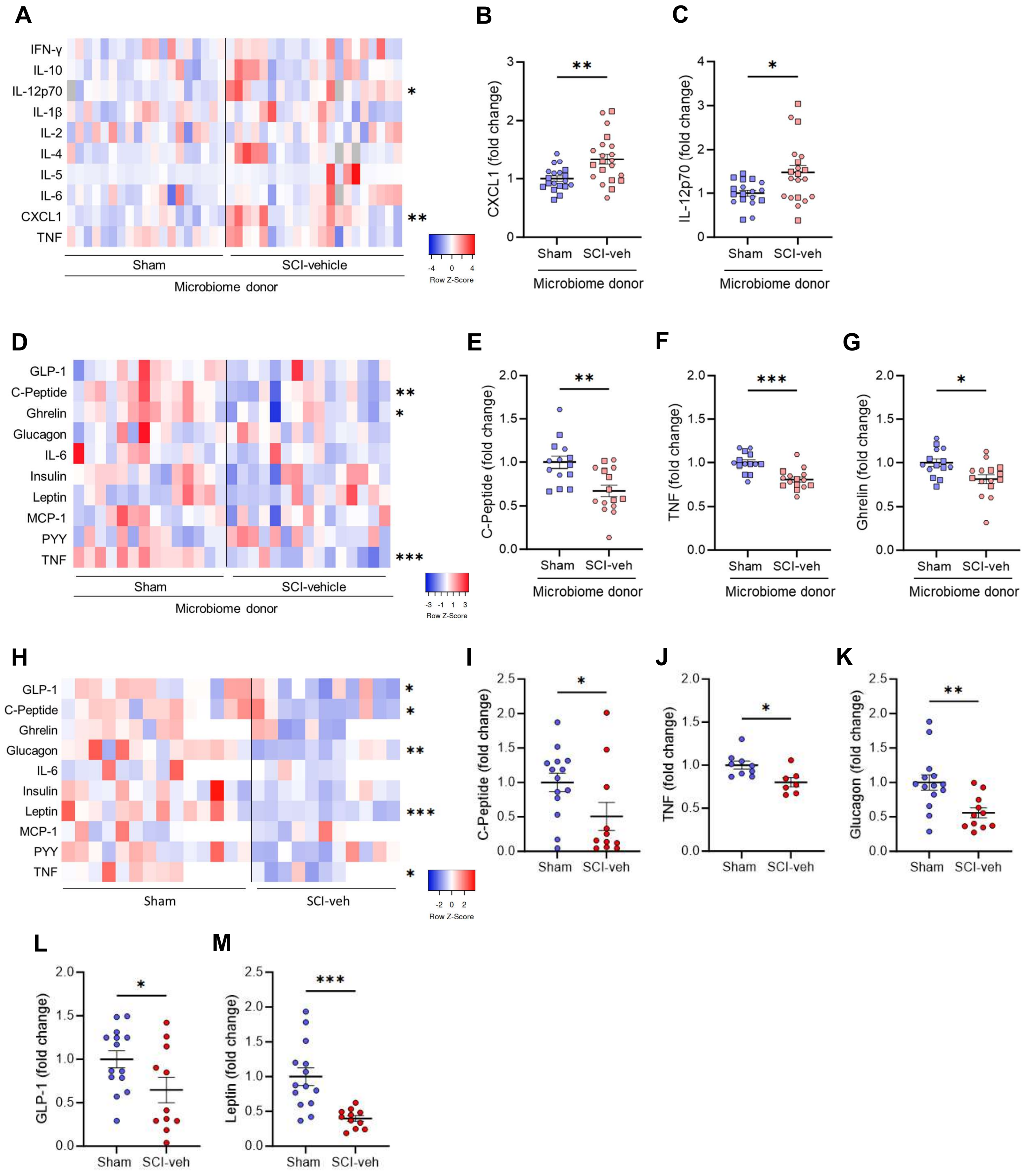

Supplementary Figure 3. SCFA-induced IL-10 signaling is necessary for SCI recovery

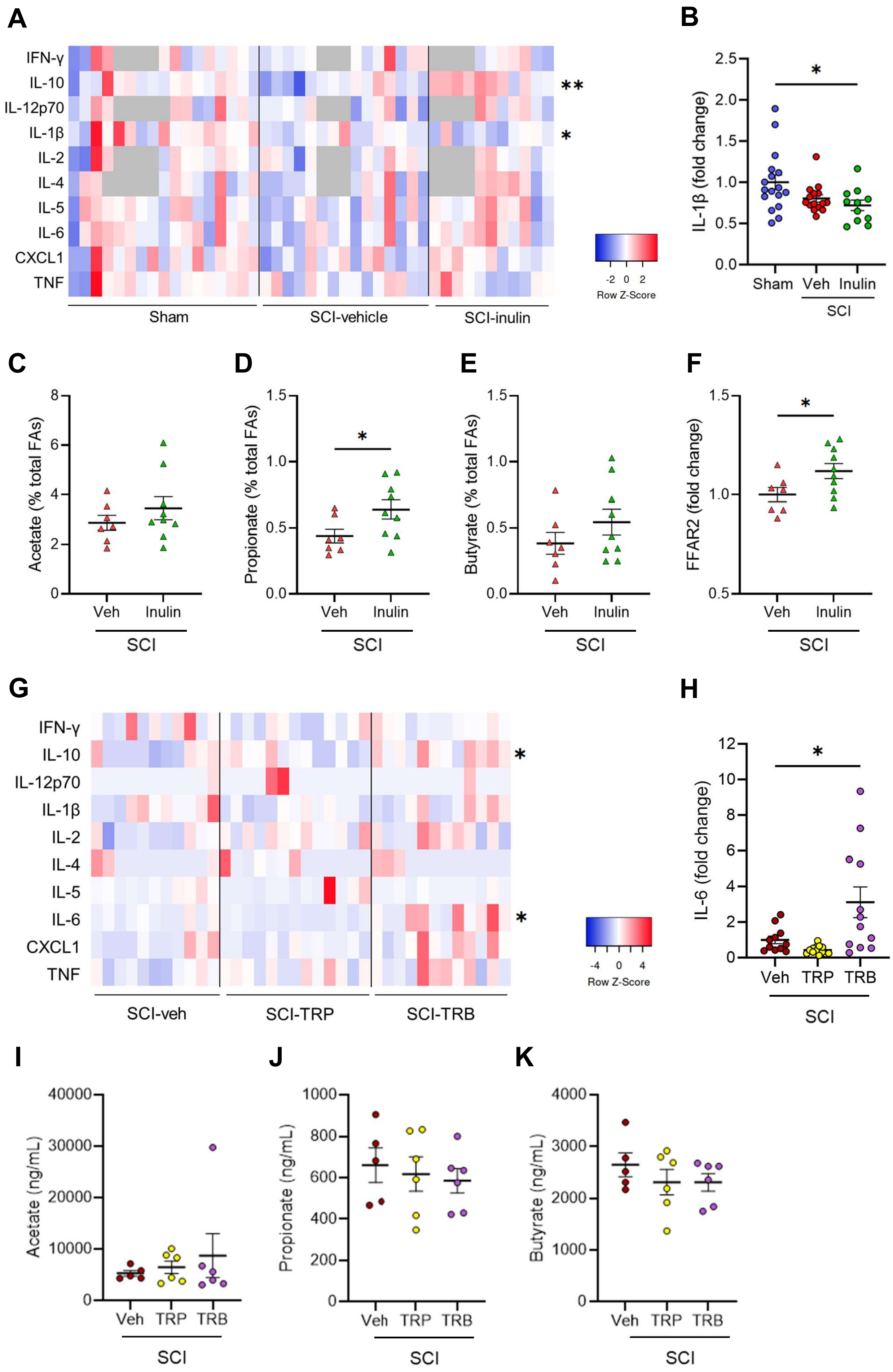

Supplementary Table 1. SCI-induced microbiome compositional differences. ASV-level taxonomic changes from stool microbiomes at 2 weeks post-SCI or sham, with 1% FDR

Endpoint			
**Sham vs SCI-Vehicle**			
**ASV**	**Sham**	**SCI-Veh**	**Adjusted *p* value (1% FDR)**
Bacteroides sp12288 (*thetaiotaomicron*)	0.0448409	0.0186638	6.94559E-07
Bacteroidales sp12656	0.156247	0.0943178	1.16422E-30
Staphylococcus lentus	0.0334159	0.00351487	1.50293E-08
Lactobacillus johnsonii	0.0577711	0.0290364	5.24441E-08
Clostridium celatum	0.0875609	0	0
Lachnospiraceae sp33565	0.00720456	0.0506877	2.82898E-16
Turicibacter sanguinis	0.124592	0.0603193	8.76368E-33
**Sham vs SCI-Inulin**			
**ASV**	**Sham**	**SCI-Inulin**	**Adjusted *p* value (1% FDR)**
Bacteroides sp12288 (*thetaiotaomicron*)	0.0448409	0.0757577	5.81151E-08
Bacteroidales sp12550	0.138146	0.028615	0
Bacteroidales sp12656	0.156247	0.188446	1.62892E-08
Staphylococcus lentus	0.0334159	0.000353782	6.74178E-09
Clostridium celatum	0.0875609	0	0
Lachnospiraceae sp33565	0.00720456	0.040268	6.73239E-09
Turicibacter sanguinis	0.124592	0	0
**SCI-Vehicle vs SCI-Inulin**			
**ASV**	**SCI-Veh**	**SCI-Inulin**	**Adjusted *p* value (1% FDR)**
Bacteroides sp12288 (*thetaiotaomicron*)	0.0186638	0.0757577	1.15921E-15
Bacteroidales sp12550	0.135412	0.028615	0
Bacteroidales sp12656	0.0943178	0.188446	2.24226E-38
Lactobacillus johnsonii	0.0290364	0.061178	5.5047E-06
Turicibacter sanguinis	0.0603193	0	2.91586E-17
**Pre-SCI vs Post-SCI**			
**ASV**	**SCI-Pre**	**SCI-2wks**	**Adjusted *p* value (1% FDR)**
Bacteroidales sp12656	0.158599	0.0943178	2.06832E-30
Lactobacillus johnsonii	0.193283	0.0290364	0
Clostridium celatum	0.022866	0	2.92759E-05
Lachnospiraceae sp33565	0.0128364	0.0506877	6.26693E-12
Turicibacter sanguinis	0.00972012	0.0603193	7.71416E-20

Supplementary Table 2. LEfSE identified taxonomic changes from stool microbiomes at 2 weeks post-SCI or sham

Bacterial taxa	Group	Effect size	*p*-value
k__Bacteria.p__Firmicutes.c__Clostridia.o__Clostridiales.f__Lachnospiraceae.g__NA.s__sp33760	SCI.Inulin	3.28014007	0.030197383
k__Bacteria.p__Firmicutes.c__Clostridia.o__Clostridiales.f__NA.g__NA.s__sp31072	SCI.Inulin	2.98922362	0.048484305
k__Bacteria.p__Proteobacteria.c__Gammaproteobacteria.o__Pseudomonadales.f__Moraxellaceae.g__Acinetobacter.s__radioresistens	SCI.Inulin	3.18049855	0.012764197
k__Bacteria.p__Firmicutes.c__Clostridia.o__Clostridiales.f__Lachnospiraceae.g__NA.s__sp32706	SCI.Inulin	3.82883697	0.01334429
k__Bacteria.p__Firmicutes.c__Clostridia.o__Clostridiales.f__Lachnospiraceae.g__NA.s__sp32638	SCI.Inulin	3.78461598	0.023383895
k__Bacteria.p__Firmicutes.c__Clostridia.o__Clostridiales.f__Lachnospiraceae.g__NA.s__sp33456	SCI.Inulin	3.17262743	0.027158625
k__Bacteria.p__Firmicutes.c__Clostridia.o__Clostridiales.f__Lachnospiraceae.g__NA.s__sp33694	SCI.Inulin	3.76036576	0.019757099
k__Bacteria.p__Firmicutes.c__Clostridia.o__Clostridiales.f__Ruminococcaceae.g__NA.s__sp34867	SCI.Inulin	3.30028204	0.027323722
k__Bacteria.p__Firmicutes.c__Clostridia.o__Clostridiales.f__Ruminococcaceae.g__Anaerotruncus.s__sp34475	SCI.Inulin	2.99522399	0.045806314
k__Bacteria.p__Proteobacteria.c__Gammaproteobacteria.o__Pseudomonadales	SCI.Inulin	3.25550068	0.016389554
k__Bacteria.p__Proteobacteria.c__Gammaproteobacteria.o__Pseudomonadales.f__Moraxellaceae.g__Acinetobacter	SCI.Inulin	3.22914314	0.016389554
k__Bacteria.p__Firmicutes.c__Clostridia.o__Clostridiales.f__Lachnospiraceae.g__NA.s__sp33417	SCI.Inulin	3.75893236	0.011625603
k__Bacteria.p__Bacteroidetes.c__Bacteroidia.o__Bacteroidales.f__Bacteroidaceae.g__Bacteroides.s__sp12288_thetaiotaomicron	SCI.Inulin	4.43785167	0.028207093
k__Bacteria.p__Firmicutes.c__Clostridia.o__Clostridiales.f__Lachnospiraceae.g__NA.s__sp33525	SCI.Inulin	3.26973262	0.048484305
k__Bacteria.p__Firmicutes.c__Clostridia.o__Clostridiales.f__Lachnospiraceae.g__NA.s__sp32701	SCI.Inulin	3.96461728	0.015562692
k__Bacteria.p__Firmicutes.c__Clostridia.o__Clostridiales.f__Lachnospiraceae.g__NA.s__sp33616_sp33639	SCI.Inulin	3.42818688	0.029407217
k__Bacteria.p__Firmicutes.c__Bacilli.o__Lactobacillales.f__Enterococcaceae.g__Enterococcus.s__faecalis	SCI.Inulin	3.05716304	0.024861262
k__Bacteria.p__Proteobacteria.c__Gammaproteobacteria.o__Pseudomonadales.f__Moraxellaceae.g__Acinetobacter.s__calcoaceticus_radioresistens_variabilis	SCI.Inulin	3.20043929	0.041388896
k__Bacteria.p__Firmicutes.c__Clostridia.o__Clostridiales.f__Ruminococcaceae.g__NA.s__sp34731	SCI.Inulin	3.43239165	0.027158625
k__Bacteria.p__Firmicutes.c__Bacilli.o__Lactobacillales.f__Enterococcaceae	SCI.Inulin	3.0725174	0.024861262
k__Bacteria.p__Bacteroidetes.c__Bacteroidia.o__Bacteroidales.f__Bacteroidaceae	SCI.Inulin	4.41723887	0.028207093
k__Bacteria.p__Bacteroidetes.c__Bacteroidia.o__Bacteroidales.f__Bacteroidaceae.g__Bacteroides	SCI.Inulin	4.45069433	0.028207093
k__Bacteria.p__Firmicutes.c__Clostridia.o__Clostridiales.f__Lachnospiraceae.g__NA.s__sp32758	SCI.Inulin	3.97272763	0.009403563
k__Bacteria.p__Firmicutes.c__Clostridia.o__Clostridiales.f__Lachnospiraceae.g__NA.s__sp32623	SCI.Inulin	3.56178608	0.041041078
k__Bacteria.p__Firmicutes.c__Clostridia.o__Clostridiales.f__NA.g__NA.s__sp31116	SCI.Inulin	3.05573343	0.04376775
k__Bacteria.p__Proteobacteria.c__Gammaproteobacteria.o__Pseudomonadales.f__Moraxellaceae	SCI.Inulin	3.23322001	0.016389554
k__Bacteria.p__Firmicutes.c__Bacilli.o__Lactobacillales.f__Enterococcaceae.g__Enterococcus	SCI.Inulin	3.06357795	0.024861262
k__Bacteria.p__Firmicutes.c__Clostridia.o__Clostridiales.f__Lachnospiraceae.g__NA.s__sp33721	SCI.Inulin	3.08898072	0.009139753
k__Bacteria.p__Firmicutes.c__Clostridia.o__Clostridiales.f__Ruminococcaceae.g__NA.s__sp35841	SCI	3.08050444	0.030197383
k__Bacteria.p__Firmicutes.c__Clostridia.o__Clostridiales.f__Lachnospiraceae.g__NA.s__sp33658	SCI	3.42184273	0.023786517
k__Bacteria.p__Firmicutes.c__Clostridia.o__Clostridiales.f__Lachnospiraceae.g__NA.s__sp32702	SCI	3.85317688	0.024861262
k__Bacteria.p__Firmicutes.c__Clostridia.o__Clostridiales.f__Lachnospiraceae.g__Lachnoclostridium.s__sp32402	SCI	3.69380202	0.034609178
k__Bacteria.p__Firmicutes.c__Clostridia.o__Clostridiales.f__Lachnospiraceae.g__NA.s__sp33739	SCI	3.15551336	0.046329809
k__Bacteria.p__Firmicutes.c__Clostridia.o__Clostridiales.f__Lachnospiraceae	SCI	5.05697495	0.048668305
k__Bacteria.p__Firmicutes.c__Clostridia.o__Clostridiales.f__Lachnospiraceae.g__NA.s__sp33565	SCI	4.28952463	0.027158625
k__Bacteria.p__Firmicutes.c__Clostridia.o__Clostridiales.f__Lachnospiraceae.g__NA.s__sp32872	SCI	3.09241069	0.028207093
k__Bacteria.p__Firmicutes.c__Clostridia.o__Clostridiales.f__Ruminococcaceae.g__Oscillibacter.s__sp34648	SCI	3.04716797	0.028937471
k__Bacteria.p__Firmicutes.c__Clostridia.o__Clostridiales.f__Lachnospiraceae.g__NA.s__sp33718	SCI	3.35285573	0.023786517
k__Bacteria.p__Firmicutes.c__Clostridia.o__Clostridiales.f__Lachnospiraceae.g__NA.s__sp32146	SCI	3.27855544	0.027158625
k__Bacteria.p__Firmicutes.c__Clostridia.o__Clostridiales.f__Ruminococcaceae.g__NA.s__sp35077	Sham	3.08269556	0.027323722
k__Bacteria.p__Firmicutes.c__Bacilli.o__Bacillales.f__Staphylococcaceae.g__Staphylococcus.s__lentus	Sham	4.22428258	0.015562692

Supplementary Table 2 (continued)			
**Bacterial taxa**	**Group**	**Effect size**	***p*-value**
k__Bacteria.p__Firmicutes.c__Erysipelotrichia.o__Erysipelotrichales	Sham	4.77308354	0.015562692
k__Bacteria.p__Firmicutes.c__Clostridia.o__Clostridiales.f__Clostridiaceae	Sham	4.65015938	0.025161049
k__Bacteria.p__Firmicutes.c__Clostridia.o__Clostridiales.f__Clostridiaceae.g__Clostridium.s__celatum	Sham	4.6301129	0.009139753
k__Bacteria.p__Firmicutes.c__Clostridia.o__Clostridiales.f__Ruminococcaceae.g__NA.s__sp35361	Sham	3.59813998	0.019660049
k__Bacteria.p__Firmicutes.c__Clostridia.o__Clostridiales.f__Ruminococcaceae.g__NA.s__sp35360	Sham	3.08921088	0.041041078
k__Bacteria.p__Proteobacteria.c__Betaproteobacteria.o__Burkholderiales.f__Alcaligenaceae	Sham	3.43800153	0.011391522
k__Bacteria.p__Firmicutes.c__Bacilli.o__Bacillales.f__Staphylococcaceae.g__Staphylococcus	Sham	4.27885163	0.037902914
k__Bacteria.p__Firmicutes.c__Clostridia.o__Clostridiales.f__Lachnospiraceae.g__NA.s__sp33419	Sham	3.32307121	0.015562692
k__Bacteria.p__Bacteroidetes.c__Bacteroidia.o__Bacteroidales.f__NA.g__NA.s__sp12550	Sham	4.72904022	0.048484305
k__Bacteria.p__Firmicutes.c__Clostridia.o__Clostridiales.f__Clostridiaceae.g__Clostridium	Sham	4.65240631	0.025161049
k__Bacteria.p__Firmicutes.c__Erysipelotrichia.o__Erysipelotrichales.f__Erysipelotrichaceae.g__Turicibacter.s__sanguinis	Sham	4.7786212	0.012764197
k__Bacteria.p__Firmicutes.c__Bacilli.o__Bacillales.f__Staphylococcaceae.g__Jeotgalicoccus	Sham	3.42659395	0.013728404
k__Bacteria.p__Firmicutes.c__Bacilli.o__Bacillales.f__Staphylococcaceae	Sham	4.30010345	0.037902914
k__Bacteria.p__Firmicutes.c__Erysipelotrichia	Sham	4.76829196	0.015562692
k__Bacteria.p__Firmicutes.c__Clostridia.o__Clostridiales.f__Lachnospiraceae.g__NA.s__sp33537	Sham	3.08288654	0.041388896
k__Bacteria.p__Firmicutes.c__Clostridia.o__Clostridiales.f__NA.g__NA.s__sp31125	Sham	3.07776853	0.041388896
k__Bacteria.p__Firmicutes.c__Clostridia.o__Clostridiales.f__NA.g__NA.s__sp31122	Sham	3.42566575	0.034411331
k__Bacteria.p__Firmicutes.c__Erysipelotrichia.o__Erysipelotrichales.f__Erysipelotrichaceae.g__Turicibacter	Sham	4.801587	0.012764197
k__Bacteria.p__Firmicutes.c__Erysipelotrichia.o__Erysipelotrichales.f__Erysipelotrichaceae	Sham	4.77876752	0.015562692
k__Bacteria.p__Firmicutes.c__Clostridia.o__Clostridiales.f__Ruminococcaceae.g__NA.s__sp35498	Sham	3.08729405	0.041388896
k__Bacteria.p__Proteobacteria.c__Betaproteobacteria.o__Burkholderiales	Sham	3.41658553	0.011391522
k__Bacteria.p__Proteobacteria.c__Betaproteobacteria	Sham	3.40401102	0.011391522
k__Bacteria.p__Firmicutes.c__Bacilli.o__Bacillales	Sham	4.30946747	0.037902914
k__Bacteria.p__Proteobacteria.c__Betaproteobacteria.o__Burkholderiales.f__Alcaligenaceae.g__Parasutterella	Sham	3.41352673	0.011391522
k__Bacteria.p__Firmicutes.c__Bacilli.o__Bacillales.f__Staphylococcaceae.g__Jeotgalicoccus.s__halophilus_halotolerans_nanhaiensis	Sham	3.4063761	0.013728404
k__Bacteria.p__Proteobacteria.c__Betaproteobacteria.o__Burkholderiales.f__Alcaligenaceae.g__Parasutterella.s__excrementihominis	Sham	3.42627509	0.011391522

Supplementary Table 3. Bacterial taxa significantly different following SCI in an independent cohort. With 1% FDR correction, alterations between Sham and SCI (a), sham and SCI-inulin (b), or SCI and SCI-inulin (c). Grey boxes indicate significant comparisons (1% FDR) between pre and post-SCI groups at 2wks post injury.

ASV	Sham	SCI-Veh	SCI-Inulin	Sig comparisons by 1% FDR	Identified in independent cohort (Fig 2)
Akkermansia muciniphila	0.032851	0.04787	0.016194	c	
Alistipes sp14336	0.029102	0.067027	0.06584	a, b	
Allobaculum sp36555	0.000152	0.021406	0.006325	a	
Bacteroidales sp12473-sp12526-sp12633	0.074217	0.084304	0.042379	b, c	
Bacteroidales sp12610	0	0.030116	0.013937	a	
Bacteroidales sp12645	0	0.019732	0.058406	b, c	
Bacteroidales sp12656	0.105666	0.052678	0.156417	a, b, c	Yes
Bacteroidales sp12768	0.033087	0.05611	0.04598	a	
Bacteroides sp12288 (thetaiotaomicron)	0.080995	0.071137	0.050251	b, c	Yes
Bifidobacterium choerinum-pseudolongum	0.049002	0.002335	0.007556	a, b	
Lachnoclostridium sp32402	0.021026	0.040619	0.01123	c	
Lachnospiraceae sp32778	0.009123	0.043795	0.014431	a, c	
Lactobacillus johnsonii	0.237838	0.079619	0.078155	a, b	Yes
Staphylococcus lentus	0.027879	0.005809	0.019373	a	Yes

Supplementary Table 4. Independent SCI-induced microbiome compositional differences. ASV-level taxonomic changes from stool microbiomes at 2 weeks post-SCI or sham, with 1% FDR, from independent cohort

Endpoint			
**Sham vs SCI-Vehicle**			
**ASV**	**Sham**	**SCI-Veh**	**Adjusted *p* value (1% FDR)**
Bifidobacterium choerinum-pseudolongum	0.0490019	0.00233502	1.38505E-15
Bacteroidales sp12610	0	0.0301156	2.27912E-07
Bacteroidales sp12656	0.105666	0.0526781	1.42237E-19
Bacteroidales sp12768	0.0330871	0.05611	7.46328E-05
Alistipes sp14336	0.0291018	0.0670273	7.71846E-11
Staphylococcus lentus	0.0278791	0.00580894	0.000146098
Lactobacillus johnsonii	0.237838	0.0796191	0
Lachnospiraceae sp32778	0.00912332	0.0437946	2.64513E-09
Allobaculum p36555	0.00015199	0.0214064	0.000254523
**Sham vs SCI-Inulin**			
**ASV**	**Sham**	**SCI-Inulin**	**Adjusted *p* value (1% FDR)**
Bifidobacterium choerinum-pseudolongum	0.0490019	0.00755608	4.74124E-08
Bacteroides sp12288 (thetaiotaomicron)	0.0809949	0.0502512	4.99359E-05
Bacteroidales sp12473-sp12526-sp12633	0.0742173	0.0423789	2.67067E-05
Bacteroidales sp12645	0	0.0584062	1.67097E-14
Bacteroidales sp12656	0.105666	0.156417	2.43458E-11
Alistipes sp14336	0.0291018	0.0658396	1.27625E-06
Lactobacillus johnsonii	0.237838	0.0781547	0
**SCI-Vehicle vs SCI-Inulin**			
**ASV**	**SCI-Veh**	**SCI-Inulin**	**Adjusted *p* value (1% FDR)**
Bacteroides sp12288 (thetaiotaomicron)	0.0711372	0.0502512	0.00013929
Bacteroidales sp12473-sp12526-sp12633	0.0843043	0.0423789	2.45024E-14
Bacteroidales sp12645	0.0197316	0.0584062	1.95902E-12
Bacteroidales sp12656	0.0526781	0.156417	0
Lachnoclostridium sp32402	0.040619	0.0112297	8.5429E-08
Lachnospiraceae sp32778	0.0437946	0.0144314	8.76893E-08
Akkermansia muciniphila	0.0478695	0.0161938	7.92208E-09
**Pre SCI vs Post SCI**			
**ASV**	**SCI-Pre**	**SCI-2wks**	**Adjusted *p* value (1% FDR)**
Other	0.0400304	0.00230997	3.01332E-20
Bifidobacterium choerinum-pseudolongum	0.0387627	0.00233502	5.35722E-19
Bacteroidales sp12473-sp12526-sp12633	0.0566063	0.0843043	1.20298E-11
Bacteroidales sp12768	0.0328508	0.05611	1.223E-08
Enterococcus faecalis	0.00133197	0.0180171	4.33168E-05
Lactobacillus johnsonii	0.130668	0.0796191	1.57727E-35
Lachnoclostridium sp32402	0.00983318	0.040619	4.98182E-14
Lachnospiraceae sp32778	0.00608032	0.0437946	3.05544E-20
Ruminococcaceae sp35297	0.0262177	0.0028927	1.11286E-08
Allobaculum sp36555	0.0653365	0.0214064	7.94526E-27

Supplementary Table 5. LEfSE identified taxonomic changes from stool microbiomes at 2 weeks post-SCI or sham, from independent cohort

Bacterial taxa	Group	Effect size	*p*-value
k__Bacteria.p__Firmicutes.c__Clostridia.o__Clostridiales.f__Lachnospiraceae.g__NA.s__sp33761	SCI	3.20746086	0.03938728
k__Bacteria.p__Firmicutes.c__Clostridia.o__Clostridiales.f__Ruminococcaceae.g__Anaerotruncus.s__sp34475	SCI	3.14434302	0.01455464
k__Bacteria.p__Bacteroidetes.c__Bacteroidia.o__Bacteroidales.f__NA.g__NA.s__sp12473_sp12526_sp12633	SCI	4.29010458	0.03319739
k__Bacteria.p__Firmicutes.c__Clostridia.o__Clostridiales.f__Lachnospiraceae.g__NA.s__sp32862	SCI	3.49736231	0.03339209
k__Bacteria.p__Firmicutes.c__Clostridia.o__Clostridiales.f__Lachnospiraceae.g__NA.s__sp32735	SCI	3.60307215	0.02671248
k__Bacteria.p__Firmicutes.c__Clostridia.o__Clostridiales.f__Lachnospiraceae.g__NA.s__sp32721	SCI	3.47470577	0.02675007
k__Bacteria.p__Firmicutes.c__Clostridia.o__Clostridiales.f__Ruminococcaceae.g__NA.s__sp33140	SCI	2.96559706	0.02508897
k__Bacteria.p__Actinobacteria.c__Actinobacteria.o__Corynebacteriales.f__Corynebacteriaceae	SCI.Inulin	3.5183703	0.01922687
k__Bacteria.p__Firmicutes.c__Clostridia.o__Clostridiales.f__Lachnospiraceae.g__NA.s__sp33731	SCI.Inulin	3.21037177	0.01440632
k__Bacteria.p__Actinobacteria.c__Actinobacteria.o__Corynebacteriales.f__Corynebacteriaceae.g__Corynebacterium	SCI.Inulin	3.51837339	0.01922687
k__Bacteria.p__Actinobacteria.c__Actinobacteria.o__Corynebacteriales	SCI.Inulin	3.51837267	0.01922687
k__Bacteria.p__Firmicutes.c__Clostridia.o__Clostridiales.f__Lachnospiraceae.g__NA.s__sp32635_sp32668	SCI.Inulin	3.09090389	0.01922687
k__Bacteria.p__Actinobacteria.c__Actinobacteria.o__Corynebacteriales.f__Corynebacteriaceae.g__Corynebacterium.s__amycolatum	SCI.Inulin	3.51837192	0.01922687
k__Bacteria.p__Firmicutes.c__Clostridia.o__Clostridiales.f__Lachnospiraceae.g__NA.s__sp33432	SCI.Inulin	3.16044082	0.01835448
k__Bacteria.p__Firmicutes.c__Clostridia.o__Clostridiales.f__Lachnospiraceae.g__Acetatifactor	SCI.Inulin	3.0869611	0.0469165
k__Bacteria.p__Firmicutes.c__Clostridia.o__Clostridiales.f__Lachnospiraceae.g__NA.s__sp33421_sp33679	SCI.Inulin	3.69414989	0.00122429
k__Bacteria.p__Firmicutes.c__Bacilli.o__Bacillales.f__Planococcaceae.g__Sporosarcina.s__luteola_pasteurii	Sham	3.15351433	0.04203087
k__Bacteria.p__Firmicutes.c__Bacilli.o__Lactobacillales.f__Lactobacillaceae.g__Lactobacillus	Sham	4.94539917	0.04846866
k__Bacteria.p__Firmicutes.c__Bacilli.o__Lactobacillales.f__Lactobacillaceae	Sham	4.93387968	0.04846866

Supplementary Table 5 (continued)

Bacterial taxa	Group	Effect size	*p*-value
k__Bacteria.p__Firmicutes.c__Bacilli.o__Bacillales.f__Staphylococcaceae.g__Staphylococcus.s__lentus	Sham	4.05241164	0.02345153
k__Bacteria.p__Firmicutes.c__Bacilli.o__Bacillales.f__Planococcaceae	Sham	3.13536277	0.04203087
k__Bacteria.p__Firmicutes.c__Erysipelotrichia.o__Erysipelotrichales.f__Erysipelotrichaceae.g__NA	Sham	3.04568574	0.00943301
k__Bacteria.p__Firmicutes.c__Erysipelotrichia.o__Erysipelotrichales.f__Erysipelotrichaceae.g__NA.s__sp36786	Sham	3.28495448	0.00107094
k__Bacteria.p__Firmicutes.c__Clostridia.o__Clostridiales.f__Lachnospiraceae.g__NA.s__sp33694	Sham	3.51794408	0.01961457
k__Bacteria.p__Firmicutes.c__Clostridia.o__Clostridiales.f__Lachnospiraceae.g__NA.s__sp33673	Sham	3.20052991	0.02425508
k__Bacteria.p__Firmicutes.c__Bacilli.o__Bacillales.f__Staphylococcaceae.g__Staphylococcus	Sham	4.23216416	0.04462127
k__Bacteria.p__Actinobacteria	Sham	4.407117	0.02774235
k__Bacteria.p__Firmicutes.c__Clostridia.o__Clostridiales.f__Lachnospiraceae.g__NA.s__sp33636	Sham	3.49964269	0.00831405
k__Bacteria.p__Firmicutes.c__Clostridia.o__Clostridiales.f__Lachnospiraceae.g__Marvinbryantia.s__sp32979	Sham	3.51749967	0.00322499
k__Bacteria.p__Firmicutes.c__Bacilli.o__Bacillales.f__Staphylococcaceae	Sham	4.22071125	0.04462127
k__Bacteria.p__Firmicutes.c__Clostridia.o__Clostridiales.f__Lachnospiraceae.g__Blautia.s__sp32038	Sham	3.5580099	0.02597101
k__Bacteria.p__Firmicutes.c__Clostridia.o__Clostridiales.f__Lachnospiraceae.g__NA.s__sp33374	Sham	3.35338706	0.02708786
k__Bacteria.p__Firmicutes.c__Clostridia.o__Clostridiales.f__NA.g__NA.s__sp31125	Sham	3.0240336	0.00502104
k__Bacteria.p__Firmicutes.c__Bacilli.o__Bacillales.f__Planococcaceae.g__Sporosarcina	Sham	3.14293813	0.04203087
k__Bacteria.p__Firmicutes.c__Clostridia.o__Clostridiales.f__Lachnospiraceae.g__NA.s__sp32622	Sham	3.84168757	0.03785401
k__Bacteria.p__Firmicutes.c__Clostridia.o__Clostridiales.f__Lachnospiraceae.g__NA.s__sp32156	Sham	3.13422797	0.03942504
k__Bacteria.p__Firmicutes.c__Clostridia.o__Clostridiales.f__Lachnospiraceae.g__Marvinbryantia	Sham	3.63670981	0.0093402

Supplementary Table 6. Reagents & Resources.

**Antibodies used**				
**Primary antibodies for Western Blot**				
**Antibody**	**Concentration**	**Host**	**Supplier**	**Cat Number**
Neuronal nitric oxide synthase (nNOS)	1:1000	Rabbit	Cell Signaling	4231S
Choline acetyltransferase (ChAT)	1:1000	Goat	Sigma	AB144P
Protein gene product 9.5 (PGP9.5)	1:1000	Rabbit	Millipore	AB1761-I
Free fatty acid receptor 2 (FFAR2 / GPR43)	1:1000	Rabbit	Thermo	PA5-111780
Glyceraldehyde 3-phosphate dehydrogenase (GAPDH)	1:1000	Rabbit	Cell Signaling	5174S
β-Actin	1:1000	Rabbit	Cell Signaling	8457S
**Secondary antibodies for Western Blot**				
**Antibody**	**Concentration**	**Host**	**Supplier**	**Cat Number**
Anti-rabbit (HRP-linked)	1:1000	Goat	Cell Signaling	7074S
Anti-goat (HRP-linked)	1:1000	Donkey	Thermo	A16005
**Primary antibodies for Immunohistochemistry**				
**Antibody**	**Concentration**	**Host**	**Supplier**	**Cat Number**
Neuronal nitric oxide synthase (nNOS)	1:100	Rabbit	Cell Signaling	4231S
Anti-HuD + HuC antibody	1:500	Rabbit	Abcam	AB184267
Protein gene product 9.5 (PGP9.5)	1:200	Mouse	Abcam	AB72911
**Secondary antibodies for Immunohistochemistry**				
**Antibody**	**Concentration**	**Host**	**Supplier**	**Cat Number**
Anti-mouse (Alexa Fluor™ 594)	1:200	Goat	Thermo	A-11005
Anti-rabbit (Alexa Fluor™ 488)	1:200	Goat	Thermo	A-11008

**ATCC Strains and Growth Conditions**				
**Bacterium**	**Medium**	**Environ ment**	**CFU per gavage**	**ATCC Number**
Lactobacillus johnsonii	de Man-Rogosa-Sharpe	Aerobic	10^16	33200
Bacteroides thetaiotaomicron	Brain-Heart Infusion	Anaerobic	10^14	29148
Clostridium celatum	Chopped Meat Carbohydrate	Anaerobic	10^10	27791
