# Supplementary material for: Diet-microbiome interactions promote enteric nervous system resilience following spinal cord injury
Source: NPJ Biofilms Microbiomes. 2024 Aug 29;10:75. doi: 10.1038/s41522-024-00556-y (PMC11362535; doi:10.1038/s41522-024-00556-y)
Supplement: Supplementary file 2 — Reporting Summary [file 41522_2024_556_MOESM2_ESM.pdf]

Corresponding author(s): Tim Sampson

Last updated by author(s): Jun 6, 2024

Reporting Summary

Nature Portfolio wishes to improve the reproducibility of the work that we publish. This form provides structure for consistency and transparency in reporting. For further information on Nature Portfolio policies, see our Editorial Policies and the Editorial Policy Checklist.

Statistics

For all statistical analyses, confirm that the following items are present in the figure legend, table legend, main text, or Methods section.

n/a	Confirmed	
☐	☒	The exact sample size (*n*) for each experimental group/condition, given as a discrete number and unit of measurement
☐	☒	A statement on whether measurements were taken from distinct samples or whether the same sample was measured repeatedly
☐	☒	The statistical test(s) used AND whether they are one- or two-sided<br>*Only common tests should be described solely by name; describe more complex techniques in the Methods section.*
☒	☐	A description of all covariates tested
☐	☒	A description of any assumptions or corrections, such as tests of normality and adjustment for multiple comparisons
☐	☒	A full description of the statistical parameters including central tendency (e.g. means) or other basic estimates (e.g. regression coefficient) AND variation (e.g. standard deviation) or associated estimates of uncertainty (e.g. confidence intervals)
☐	☒	For null hypothesis testing, the test statistic (e.g. *F*, *t*, *r*) with confidence intervals, effect sizes, degrees of freedom and *P* value noted<br>*Give P values as exact values whenever suitable.*
☒	☐	For Bayesian analysis, information on the choice of priors and Markov chain Monte Carlo settings
☒	☐	For hierarchical and complex designs, identification of the appropriate level for tests and full reporting of outcomes
☒	☐	Estimates of effect sizes (e.g. Cohen's *d*, Pearson's *r*), indicating how they were calculated

Our web collection on statistics for biologists contains articles on many of the points above.

Software and code

Policy information about availability of computer code

Data collection	IHC sides were imaged with a Leica SP8 multiphoton confocal microscope with Leica Application Suite software. Gut contractility recordings were collected using AxoClamp software.
Data analysis	GraphPad Prism 10 statistics software was used for all analyses, ImageJ (FIJI) was used to analyze western blot and IHC imaging data, ClampFit was used to analyze gut contractility recording data.

For manuscripts utilizing custom algorithms or software that are central to the research but not yet described in published literature, software must be made available to editors and reviewers. We strongly encourage code deposition in a community repository (e.g. GitHub). See the Nature Portfolio guidelines for submitting code & software for further information.

Data

Policy information about availability of data

All manuscripts must include a data availability statement. This statement should provide the following information, where applicable:

- Accession codes, unique identifiers, or web links for publicly available datasets
- A description of any restrictions on data availability
- For clinical datasets or third party data, please ensure that the statement adheres to our policy

All raw numerical data, statistical outputs, western blots are available in the Source Data files associated with this manuscript. 16S sequencing files are uploaded and available via the NCBI Sequence Read Archive. Analyzed microbiome files and gut contractility files are available on Zenodo. Accession numbers available in

manuscript.

Research involving human participants, their data, or biological material

Policy information about studies with human participants or human data. See also policy information about sex, gender (identity/presentation), and sexual orientation and race, ethnicity and racism.

Reporting on sex and gender	N/A
Reporting on race, ethnicity, or other socially relevant groupings	N/A
Population characteristics	N/A
Recruitment	N/A
Ethics oversight	N/A

Note that full information on the approval of the study protocol must also be provided in the manuscript.

Field-specific reporting

Please select the one below that is the best fit for your research. If you are not sure, read the appropriate sections before making your selection.

☒ Life sciences ☐ Behavioural & social sciences ☐ Ecological, evolutionary & environmental sciences

For a reference copy of the document with all sections, see nature.com/documents/nr-reporting-summary-flat.pdf

Life sciences study design

All studies must disclose on these points even when the disclosure is negative.

Sample size	No statistical sample size calculations were performed prior to experiments. Sample size was based on prior publications in the field.
Data exclusions	Mice with SCI who presented with a BMS score of 2 or higher one day post-injury were excluded from the study due to insufficient injury. Sham mice who presented with BMS below 9 at 1-DPI were considered injured and were excluded. Gnotobiotic mice were excluded from the study if fecal pellet culture or qPCR identified bacterial contamination at the experimental endpoint. Monocolonized mice with multiple species by qPCR or selection plating or germ-free mice with any detectable bacteria in stool at their endpoints were excluded from all analyses.
Replication	Each experiment is compiled of data pooled from multiple, independent cohorts of animals as indicated in the figure legends and source data.
Randomization	Mice were randomly allocated into experimental groups prior to surgeries or bacterial manipulation.
Blinding	Investigators were generally not blinded to experimental groups, due to the highly visible nature of SCI. However, end-point BMS scoring and IHC imaging and analyses were performed by blinded investigators. Data collected by core facilities, as indicated in the methods, were always blinded, including cytokine measurements, metabolite measurements, and microbiome sequencing & analysis.

Reporting for specific materials, systems and methods

We require information from authors about some types of materials, experimental systems and methods used in many studies. Here, indicate whether each material, system or method listed is relevant to your study. If you are not sure if a list item applies to your research, read the appropriate section before selecting a response.

Materials & experimental systems

n/a	Involved in the study	
☐	☒	Antibodies
☒	☐	Eukaryotic cell lines
☒	☐	Palaeontology and archaeology
☐	☒	Animals and other organisms
☒	☐	Clinical data
☒	☐	Dual use research of concern
☒	☐	Plants

Methods

n/a	Involved in the study	
☒	☐	ChIP-seq
☒	☐	Flow cytometry
☒	☐	MRI-based neuroimaging

Antibodies

Antibodies used	Neuronal nitric oxide synthase (Cell Signaling, 4231S), Choline acetyltransferase (Sigma, AB144P), Protein gene product 9.5 (Millipore, AB1761-I), Free fatty acid receptor 2 (Thermo, PA5-111780), Glyceraldehyde 3-phosphate dehydrogenase (Cell Signaling, 5174S), β-Actin (Cell Signaling, 8457S), Anti-rabbit, HRP-linked (Cell Signaling, 7074S), Anti-goat, HRP-linked (Thermo, A16005), Protein gene product 9.5 (Abcam, AB72911), Anti-mouse, Alexa Fluor™ 594 (Thermo, A-11005), Anti-rabbit, Alexa Fluor™ 488 (Thermo, A-11008). Anti-HuD + HuC antibody (Abcam, AB184267).
Validation	Antibody validations and citations are available on manufactures websites, listed beside each antibody below.<br>Neuronal nitric oxide synthase (Cell Signaling, 4231S): https://www.cellsignal.com/products/primary-antibodies/nnos-c7d7-rabbit-mab/4231?_requestid=988551<br>Choline acetyltransferase (Sigma, AB144P): https://www.sigmaaldrich.com/US/en/product/mm/ab144p<br>Protein gene product 9.5 (Millipore, AB1761-I): https://www.emdmillipore.com/US/en/product/Anti-Protein-Gene-Product-9.5-Antibody,MM_NF-AB1761-I<br>Free fatty acid receptor 2 (Thermo, PA5-111780): https://www.fishersci.com/shop/products/gpr43-polyclonal-antibody-invitrogen-5/PIPA5111780<br>Glyceraldehyde 3-phosphate dehydrogenase (Cell Signaling, 5174S): https://www.cellsignal.com/products/primary-antibodies/gapdh-d16h11-xp-rabbit-mab/5174<br>β-Actin (Cell Signaling, 8457S): https://www.cellsignal.com/products/primary-antibodies/b-actin-d6a8-rabbit-mab/8457<br>Anti-rabbit, HRP-linked (Cell Signaling, 7074S): https://www.cellsignal.com/products/secondary-antibodies/anti-rabbit-igg-hrp-linked-antibody/7074<br>Anti-goat, HRP-linked (Thermo, A16005): https://www.thermofisher.com/antibody/product/Donkey-anti-Goat-IgG-H-L-Cross-Adsorbed-Secondary-Antibody-Polyclonal/A16005<br>Protein gene product 9.5 (Abcam, AB72911): https://www.abcam.com/products/primary-antibodies/pgp95-antibody-bh7-ab72911.html<br>Anti-mouse, Alexa Fluor™ 594 (Thermo, A-11005): https://www.thermofisher.com/antibody/product/Goat-anti-Mouse-IgG-H-L-Cross-Adsorbed-Secondary-Antibody-Polyclonal/A-11005<br>Anti-rabbit, Alexa Fluor™ 488 (Thermo, A-11008): https://www.thermofisher.com/antibody/product/Goat-anti-Rabbit-IgG-H-L-Cross-Adsorbed-Secondary-Antibody-Polyclonal/A-11008<br>Anti-HuD + HuC antibody (Abcam, AB184267): https://www.abcam.com/products/primary-antibodies/hud--huc-antibody-epr19098-ab184267

Animals and other research organisms

Policy information about studies involving animals; ARRIVE guidelines recommended for reporting animal research, and Sex and Gender in Research

Laboratory animals	8-10wk old wild-type C57Bl6/J, IL10rb-/-, and DBA/2J mice were obtained from Jackson Laboratory (#000664, 005027, & 000671). Germ-free DBA/2NTac mice were obtained from Taconic Biosciences (#DBA2).
Wild animals	N/A
Reporting on sex	Male mice were used for all SCI surgeries. Male and female germ-free DBA2 mice were used in recolonization studies.
Field-collected samples	N/A
Ethics oversight	Experimental procedures were approved by the Institutional Animal Care and Use Committee of Emory University (Protocols 201700855, 201900030, and 201900145).

Note that full information on the approval of the study protocol must also be provided in the manuscript.
